# Transitioning to a Data Driven Mental Health Practice: Collaborative Expert Sessions for Knowledge and Hypothesis Finding

**DOI:** 10.1155/2016/9089321

**Published:** 2016-08-17

**Authors:** Vincent Menger, Marco Spruit, Karin Hagoort, Floor Scheepers

**Affiliations:** ^1^Department of Information and Computing Sciences, Utrecht University, P.O. Box 80089, 3508 TB Utrecht, Netherlands; ^2^Department of Psychiatry, University Medical Center Utrecht, P.O. Box 85500, 3508 GA Utrecht, Netherlands

## Abstract

The surge in the amount of available data in health care enables a novel, exploratory research approach that revolves around finding new knowledge and unexpected hypotheses from data instead of carrying out well-defined data analysis tasks. We propose a specification of the Cross Industry Standard Process for Data Mining (CRISP-DM), suitable for conducting expert sessions that focus on finding new knowledge and hypotheses in collaboration with local workforce. Our proposed specification that we name CRISP-IDM is evaluated in a case study at the psychiatry department of the University Medical Center Utrecht. Expert interviews were conducted to identify seven research themes in the psychiatry department, which were researched in cooperation with local health care professionals using data visualization as a modeling tool. During 19 expert sessions, two results that were directly implemented and 29 hypotheses for further research were found, of which 24 were not imagined during the initial expert interviews. Our work demonstrates the viability and benefits of involving work floor people in the analyses and the possibility to effectively find new knowledge and hypotheses using our CRISP-IDM method.

## 1. Introduction

With the increase of the amount of data that is collected and stored in many different fields in this digital age, the amount of data that is being collected in health care has also increased enormously over the past years. Electronic Patient Record (EPR) software has become more widespread in hospitals and other health care institutions, both worldwide and in Netherlands [1, 2]. This central approach to patient data management enables using patient data for clinical research purposes through various data mining techniques [3, 4].

Where Randomized Controlled Trials are the current gold standard in mental health research, the sample size and lack of selection bias of a data analysis approach can offer a novel and substantial contribution to mental health research by discovering complex interaction patterns of cause and outcome, resilience and vulnerability, and risk and outcome [5]. In previous studies, data driven research has led to new scientific knowledge, improvement of patient treatment, and reduction in administrative load or financial savings, both in general health care [6] and in mental health care [7–9].

Although these approaches are undeniably beneficial, they are hypothesis driven and therefore do not necessarily exploit the full potential of data driven research. Data analysis enables the possibility of uncovering relations, patterns, and trends that were previously neither expected nor hypothesized [10–12]. Generated hypotheses can subsequently lead to replication studies with higher probability of success, because of their groundedness in the data [13]. This exploratory approach comes with its own challenges, yet it also appears to have great benefits for the health care domain [4].

Another possible benefit of this exploratory approach is the ability to involve local professionals and patients in the analysis process. Where currently much of the data analysis literature in health care focuses on the technical aspects of the transition to a more data driven standard (e.g., [14]) transforming the workforce mindset and business processes in daily practice is a challenge that is not to be underestimated [15]. The staff that has to work with the outcomes of a data analysis project is usually unfamiliar with the concept of data analysis, which creates a gap between domain experts and the technical staff performing the analysis [16, 17]. Moreover, not involving clinical practitioners in the project will lead to them feeling surpassed, which will lead to a failure to adopt the technology and thus a failure of the project. A high amount of interaction with the local workforce during a data analysis project will mitigate this problem, by constantly requiring their input in determining relevant topics and outcomes [18]. Cooperation with domain experts will both strengthen the analysis and pave the way for an easy implementation of results that are eventually discovered.

In an effort to benefit from the opportunities of data driven research and to simultaneously tackle the challenges of overcoming the gap between domain experts and technical staff, we report on an interactive data analysis project that focuses on collaborative knowledge and hypothesis finding in the psychiatry department of the University Medical Center Utrecht in Netherlands. This work contributes to the field of data analysis in mental health care by exploring the benefits and limitations of a predominantly nonhypothesis driven approach that actively involves the local workforce in the process and in that way helps mental health care institutions transition to a more data driven practice. This specific interactive data analysis approach that we have pursued has not been applied before in the context of mental health.

## 2. Materials and Methods

To investigate the possibilities and limitations of an interactive data analysis project as described in Introduction, we conducted a single exploratory case study [19] at the psychiatry department of the University Medical Center Utrecht (UMCU) in Netherlands. The goal of the case study is to explore the benefits and limitations of a data analytics project that

1. focuses on finding new knowledge and hypotheses,
2. involves local health care professionals in every step of the process.

After researching and comparing the Cross Industry Standard Process for Data Mining (CRISP-DM) [20], Knowledge Discovery in Databases (KDD) [21], Sample Explore Modify Model Assess (SEMMA) [22], and 3 Phases' Method (3PM) [23], CRISP-DM was selected to structure the case study. CRISP-DM is a well-defined process that is widely adopted in the industry [24]. Moreover, it emphasizes the organizational part of data mining, which is in line with our project goals. CRISP-DM consists of the following six steps: domain understanding, data understanding, data preparation, modeling, evaluation, and deployment.

**Figure 1:**
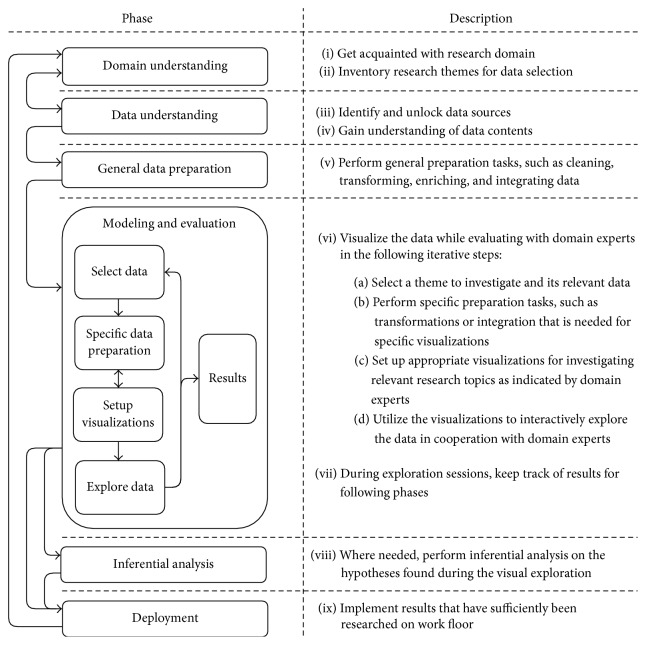
Overview of the CRISP-IDM method: a CRISP-DM based process for interactive data mining.

For making the CRISP-DM suitable for doing the exploratory data analysis that also involves health care professionals in every step of the process, we propose the following three modifications. First, we aggregate the modeling and evaluation phases into one iterative phase, that requires collaboration with domain experts. To enable this, data visualization will be used as a modeling tool, allowing participation for those unfamiliar with data analysis. Second, we distinguish between the general and the specific preparation phases, in which, respectively, generic preparation tasks and tasks that are revealed during the exploratory modeling and evaluation phase are carried out. Third, an optional inferential analysis step is added that in many cases will be necessary to be able to bring exploratory analysis results or generated hypotheses to the daily practice with sufficient confidence. An overview of the approach process is depicted in Figure 1, within the left column the different phases and their relation and in the right column the most important goals of each phase. This overview can be seen as a specification of CRISP-DM to make it applicable to doing exploratory and interactive data analysis, which we named CRISP-IDM: Cross Industry Standard Process for Interactive Data Mining. While we have applied this approach in the context of mental health care, it can easily be applied to other domains as well, since it is generic and relies on local domain experts and data sources.

From April 2015 through November 2015, the case study was implemented along the phases of our proposed CRISP-IDM method.

### 2.1. Domain Understanding

The case study was conducted at the psychiatry department of the UMCU. It consists of four units that specialize in severe affective disorders, psychotic disorders, developmental disorders, and urgent care. There are roughly 60 inpatient treatment positions. Combined with outpatient treatment, this results in approximately 2000 unique patients annually. Relevant clinical staff consists mainly of psychiatrists, psychologists, nurses, and additional staff that directly support treatment such as therapists and social workers. The department has a secondary care function regionally, as well as a tertiary care function nationally. This entails the presence of a diverse population, including patients that have been referred by general practitioners or other primary care institutions, patients that require urgent care, and patients with more complex symptomatology. As a university medical center, the UMCU is assigned to conduct research, which makes this center suitable for carrying out the case study.

In order to become acquainted with the psychiatry department, semistructured interviews were conducted with six different representatives. This included a psychiatrist of each of the four psychiatry units, a board level psychiatrist, and a psychologist. According to Yin [19] semistructured interviews are appropriate when exploring a new topic. The initial questions were open questions pertaining to the data that is recorded within the psychiatry department and possible research directions with this data. Interviews were transcribed afterwards. Although the data mining project is explicitly nonhypothesis driven, that is, not intended to answer specific research questions, interviews with health care professionals resulted in a clear mapping of specific themes that are needed for selecting relevant data sources and general data preparation. From these six interviews, a total of 28 topics were identified using Inductive Content Analysis (ICA) [25]. Subsequently, this list of topics was further categorized into seven themes, again using ICA. In a follow-up meeting with five of the six interviewed health care professionals, both the individual topics and the higher level themes were rated on their relevance, resulting in a prioritization of both. The five most relevant topics of the 28 are displayed in Table 1, and the themes they are categorized into in Table 2. Once again, the reason for identifying the topics is not that they must be researched during the project, but for providing general insight into the type of analyses that can be conducted; for guiding the analysis only the higher level themes were used. The prioritization was determined by the number of times a topic was judged to be of clinical relevance by experts under constrained selection, and for the themes in Table 2 this was conducted in the same way.

The themes 2 and 3 in Table 2 show a direct relation to the daily practice of health care professionals. The admission and dismissal theme mostly pertains to questions regarding the length of admission and the likeliness of readmission, while the aggression theme concerns questions about aggression incidents that occur with local inpatients. The context factors (theme 1) and patient referrals (theme 4) themes however mostly relate to the part of the care process that occurs outside of the UMCU, for example, the social and economic background of a patient and the continuing of a treatment in other institutions. Themes 2 and 3 therefore require data that is locally generated and stored, and themes 1 and 4 require external data that can be connected to the local data.

### 2.2. Data Understanding

Researching the identified research themes in Table 2 requires selection of relevant data sources, gaining access to these data sources, and unlocking their relevant data. Within a health care institution multiple possible data sources usually exist, such as an Electronic Patient Record (EPR), lab measurements, imaging data and other databases that contain relevant information [26]. Externally, census data, geographical data, and data gathered by other care institutions contain information relevant for analysis. These external data sources are important for doing analysis concerning the environmental factors that interplay with development and treatment of symptoms, as well as comparing the patient group of the UMCU with healthy citizens or patients in other care institutions.

Lab measurements and imaging data were omitted due to few mentions during the domain understanding phase and limited amounts of available data on our patient population. It was not feasible to obtain data from other care institutions within the duration of the project, due to privacy constraints and limited resources. The ultimately accessible data sources and their data are listed in Table 3. The various entities are representative of all important care process aspects and allow performing analyses in all themes except patient referrals. For the sake of enabling exploratory data analysis, all variables were initially included for each of the datasets. The purpose of the exploratory analysis is finding new and unexpected relations or patterns, so naturally no variables should be excluded at the outset.

From the Electronic Patient Record, several data entities were available:

1. All patients receive a diagnosis according to the standard Diagnostic and Statistical Manual of Mental Disorders IV (DSM-IV) classification system on four axes (1) primary diagnostic for treatment, (2) personality disorders, (3) medical or physical disorders, and (4) psychosocial and environmental factors.
2. When starting treatment, a treatment plan is written by a health care professional, in which the type and duration of treatment are described, along with free text fields describing the symptoms and background of a patient.
3. During treatment, patients may have one or more medication prescriptions, which include the type, dose, and frequency of medication, the period in which the medication is prescribed, and possible mutations.
4. For the purpose of monitoring the state of a patient, Routine Outcome Monitoring (ROM) is performed by scoring a questionnaire or metric on a certain time interval. Available ROM methods include Health of the Nation Outcome Scales (HONOS), Kennedy Axis V, Child Behaviour Checklist (CBCL), and the Global Assessment of Functioning (GAF). These all measure another aspect of the well-being of a patient and are all taken at different moments in the care process with different time intervals.
5. Admission information comprises, for example, the date of admission and dismissal, to which unit the patient was admitted, and the type of admission.
6. Free text reports are written by psychiatrists and nurses about their admitted patients during each of the three shifts on a day. These unstructured text fields contain information on the daily well-being and activities of a patient.

When an aggression incident occurs, mandatory reporting takes place in a separate Incident Reporting System (IRS). For each incident, several structured variables are recorded, such as the patients and staff involved, the location and time of the incident, and type of aggression. Additionally, in free text variables information about the events leading up to the incident and the incident itself are captured.

Externally, open data from the Dutch national census bureau was acquired, such as statistics about the average income, urbanization, and type of homes in the living environment of the patient. From a geographical source, more detailed data about the amount of green space in the direct vicinity of patients was obtained.

### 2.3. Data Preparation

Since the available data is stored in a way that is not intended for doing research, preprocessing on the data was necessary to convert it to an appropriate format for exploratory analysis. The most important tasks in preparing the data are transforming, cleaning, integrating, reducing, and discretizing the data [27]. Since in the exploratory, nonhypothesis driven type of approach not all data preparation steps are known in advance, at first only the general tasks that can be carried out without knowing specific modeling goals were performed. We therefore call this phase the general data preparation phase and iteratively carry out the specific data preparation in the modeling and evaluation phase.

First of all, the data transformation consists of simple tasks such as parsing date variables and changing variable names. Many categoric fields do not contain data that is understandable for a user by default but instead contains codes or abbreviations; these fields were transformed to be user readable. In this stage of the process, no transformations were done with regard to incomplete data, because the modeling software that is discussed in Section 2.4 is designed to handle this. For textual data, several transformations such as stemming and stop word removal were applied, to achieve both data reduction and noise reduction.

After transformation, data was cleaned, for example, by removing redundant variables and duplicate records. This also includes identification variables and metadata that are meaningful in the system the data was sourced from, but not in the context of analysis.

Finally, to be able to make statements about concepts that are captured in different datasets, datasets that were often mentioned in relation to each other in the expert interviews were integrated. Additionally, in this step the data was enriched by using open data from both the Diagnostic and Statistical Manual of Mental Disorders (DSM) and the Anatomical Therapeutic Chemical Classification System (ATC). This allows easier patient selection by looking at diagnoses and medication as a hierarchy, for example, distinguishing the anatomical main group level and the chemical substance level of a drug.

### 2.4. Modeling and Evaluation

To be able to involve the workforce in the modeling and evaluation phases, as well as doing research in an exploratory way, a different approach than the traditional one is required. In a usual setting, a team of data analysts or technical experts receive a specific problem that is posed by domain experts, use technical or statistical modeling software to answer the problem, and then collaborate with the domain experts again to evaluate and possibly refine the models. This setting is not appropriate for our proposed approach, both because our approach is not aimed at answering specific questions but at exploring the data to find new knowledge and hypotheses and because our approach aims to include health care professionals in every step of the process. Therefore an interactive data visualization tool is used to model the data. This tool enables direct feedback from health care professionals who can be present and directly participate in the modeling process in an approachable way.

The visualization tool supports several types of visualizations, ranging from basic to advanced. Basic visualizations, for example, include scatter plots, histograms, and map plots. More advanced examples are network visualizations, bar charts, trend lines, and word walls that enable visualization of text variables. Furthermore, the tool supports a wide range of selection and drilling down options that easily enables zooming in on specific parts of the patient population. Most notably, the tool has an interactive modus operandi, which supports real time updating of selection sets in multiple visualizations and therefore suits well with our goal of domain expert participation with direct feedback. This method excels in its visual interface and interactive nature, as opposed to traditional data mining tools which usually center around programming code and textual or numeric output.

**Figure 2:**
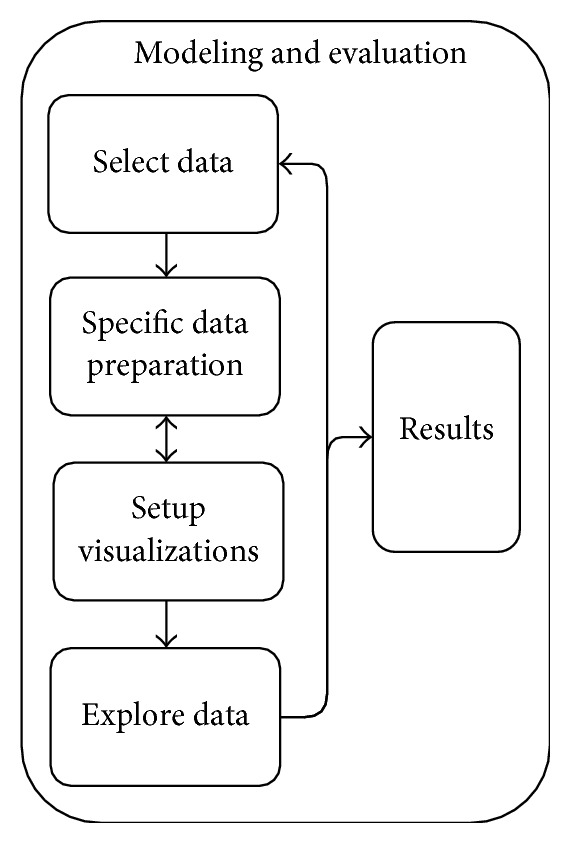
The modeling and evaluation phase process in CRISP-IDM.

The modeling and evaluation phase is done in weekly sessions that require the presence and collaboration of both health care professionals and technical staff. The health care professionals are needed to guide the analysis and find new interesting visualizations with their expertise of the domain, and the technical staff is only needed to facilitate the analysis by introducing the data and visualizations used and operating the software. Note that the exploration was guided by the domain experts and only facilitated by the technical staff, since they lack knowledge of the psychiatry domain. The weekly sessions were held 3-4 consecutive times for each of the identified themes in Table 2. The process consists of five iterative steps (Figure 2):

1. In the data selection step, initially a small set of data relevant to the current theme was selected. In next iterations, additional data was added to this set, whenever indicated to be relevant by the health care professionals.
2. In the specific preparation step, preparation that was not done in the general step is carried out where necessary for specific purposes. This includes integrating different data entities that were not linked in the source data and data transformations or derivation of variables that were needed for specific visualizations requested by present health care professionals.
3. The visualization setup step constituted loading the prepared data into the visualization tool and initially creating simple, descriptive visualizations of the data at hand, such as the number of admissions over time, distributions of the diagnoses, and most prescribed medications. In next iterations, more complex visualizations based on practitioner input were added.
4. During the exploration step health care professionals explored the data, supported by technical staff. The exploration was guided by the health care professionals, for example, by selecting patient population subgroups based on their different characteristics, comparing trends over time, and looking for patterns in the visualizations. The technical staff facilitates this analysis, by performing the actions that are requested by the health care professionals and by helping interpret the visualizations. The collaboration during the session enables a creative process that leads to new ideas for visualizations and the relating of new concepts and datasets by domain experts. These were explored in the next exploration cycle, because they often required additional data and preparation that was not yet performed in steps (1)–(3). Steps (1)–(4) are therefore iterative, since not all visualizations are initially known; they are a result of the creative process of looking at the data in collaboration.
5. The constant visual feedback of the modeling process enabled direct observation of which visualizations depict interesting results, such as possible correlations, textual terms that stand out or trends in bar charts. The products of steps (1)–(4) are these kinds of results that require action, that is, relations that can visually be confirmed and require further research. These observations are the direct result of the modeling and evaluation phase and were noted for following phases of the CRISP-IDM process.

As an example, during the iterations of the aggression theme, a peak in aggression incidents was found to exist on the fifth day of admission. Initially, a small set of data was selected for the aggression theme, including the aggression and the diagnosis datasets. During the first iteration, only some descriptive statistics on aggression were visualized, such as the number of incidents per unit and the most occurring types of aggression (e.g., verbal, physical). Experts then indicated that they were interested to see if there was any change in the number of incidents over time. This visualization was added in the next iteration but did not show any interesting results. Another expert wondered if it was more likely for an incident to happen at the beginning of the end of an admission. In the next iteration, admission data was therefore added and integrated with the aggression data. Visualization showed a peak in incidents on the fifth day of admission (Figure 7), which domain experts did not expect and judged to be unknown. This peak in incidents was therefore noted as a result for further inspection during the following phases. Other results and their strength are further elaborated upon in Section 3.

In a period of three months, a total of 19 interactive exploration sessions with two or three domain experts and at least one technical expert present were conducted. A total of 18 health care professionals were involved in exploring the data, and each expert attended 2.3 times on average. A breakdown of the number of people and attendances is visible in Table 4, where it can also be verified that professionals from each part of the psychiatric department process were included in the sessions.

### 2.5. Deployment

**Figure 3:**
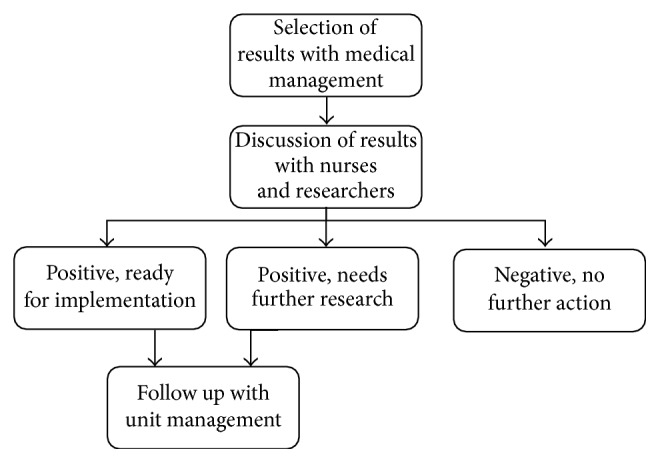
The deployment phase process in CRISP-IDM.

The deployment phase typically focuses on implementing the results that were obtained in the modeling phase on the work floor. This part of the process was in our case not very distinct from a typical data mining project, since results that were obtained and confirmed needed to be transformed to daily work practice. This concerns both results that were judged to be strong enough during the visual modeling, as well as results that were additionally tested using inferential analysis. The process that describes how results are deployed is depicted in Figure 3.

Initially, a selection of results that can contribute to daily work practice was determined with the help of medical management, including the board and the leading psychiatrists of each of the four units in the psychiatry department. After a first rough selection is made, these results were discussed with relevant nursing staff and local researchers or policymakers. Incorporating both board and management level professionals as well as daily workforce ensured that eventually implemented results are widely supported in the department. Discussing the results with workforce can lead to the agreement that an outcome is not suitable for implementation, in which case no further action is required, or a positive response. In this case, the result is either ready for implementation or needs further research, such as connecting the result to relevant literature or exploration of other data sources or the electronic patient file to investigate the result on a higher level. Depending on the type of result, further actions were determined and assigned in a follow-up with the management of the relevant unit(s).

## 3. Results and Discussion

**Figure 4:**
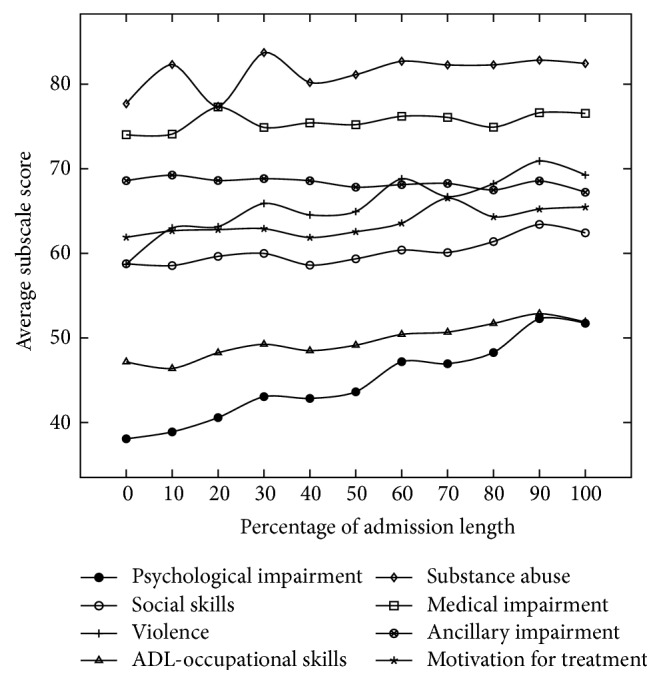
The average score of the eight Kennedy Axis V subscales over time, with the *x*-axis representing the percentage of the total admission length (429 admissions, 2531 Kennedy Axis V reports). It can be seen that except for the Psychological Impairment subscale, the variation during the admission is minimal. Domain experts indicated that the amount of variation that can be seen does not justify scoring the Kennedy Axis V on a regular basis.

**Figure 5:**
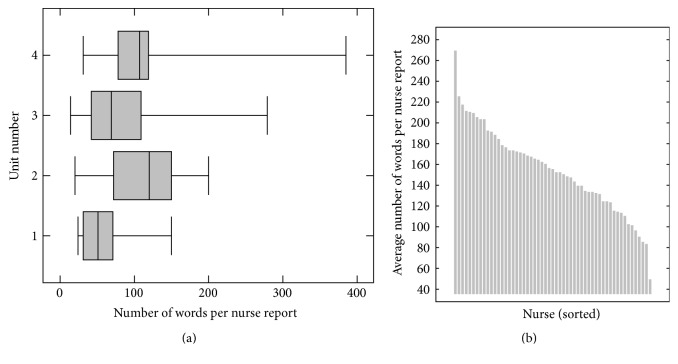
On the left (a), a box plot for each of the four units can be seen that shows the variation of the number of words in a nurse report (140,719 reports). It can be seen that there are substantial differences among the units, for instance, by looking at the median number of words in a nurse report: for Unit 1 this is around 50, but for Unit 2 this is around 120. On the right (b), the average status report length per nurse for units 3 is depicted (33,418 reports). Between different nurses, large differences can occur. Other units show very similar distributions. In both images, only nurses that wrote at least 50 statuses were included in the analysis.

Two of the results that were found during the modeling phase were implemented on the psychiatry work floor:

1. The Kennedy Axis V, a specific Routine Outcome Monitoring method that is used in all four units of the psychiatry department, scores the well-being of a patient in eight areas on a 0–100 scale and additionally enables nurses to enter a note or explanation in a text field. The numeric scores are supposed to reflect the current state of a patient's well-being on regular intervals (e.g., weekly) during an admission. Data analysis however shows that for seven of the eight subscales the score remains almost constant during an admission (Figure 4), thus invalidating the need to score it on a regular basis. As a result, the numeric scores are filled in less frequently, yielding time savings and lower administrative load at the work floor.
2. The nurse reports that are written about admitted patients during each of the three daily shifts were shown to be of varying extensiveness, both among different nurses and among different units within the department (Figure 5). Among health care professionals there was a broad agreement that good reports are concise, so that the writing is not time consuming and that readers of the reports can quickly recognize key points. This has resulted in a new guideline for writing nurse reports, again leading to a decreased administrative burden for nurses.

In both cases, the fact that the people who have to work with the outcomes of the project aided in finding these outcomes paved the way for an easy implementation of these results.

During the modeling and evaluation phase some limitations of the interactive visualization software came to light as well. First, although the interactive visualization software used allows easy participation for nontechnical staff, this feature comes with a trade-off in methodological rigor. For example, the software does not allow statistical testing or more sophisticated data analysis tasks. This kind of advanced analysis is more challenging to conduct in the presence of health care professionals because it usually centers around numerical output instead of visualization, which is more difficult to understand for clinicians. At times a correlation, pattern, or trend appeared to exist in a visualization, yet a decisive answer cannot be given without reverting to more traditional statistical software.

Second, while reverting to the statistical software can in many cases determine the strength of a result with a correlation coefficient or a *p* value, the fact that many other relationships between variables have been discarded without any testing makes this an unusual instance of the multiple comparisons problem. Even if a statistically significant relation is found, the fact that many relations have been manually examined without any testing weakens the result. Although this effect is usually stronger in machinal computation of many correlations, it is definitely present here, and it prohibits simply viewing the outcome of statistical test as a direct result. The exploratory character of the expert sessions therefore limits the ability to conclusively verify results, and in many cases additional research, for example, on other similar datasets, is required.

For the reasons mentioned above, other results that have been found in the data are classified as hypotheses for further investigation, rather than direct results. Put differently, the exploratory outcomes provide the input for inferential analysis. The two implemented results differ from these hypotheses in that the data describes the entire population, and a statistical method is not indispensable to validate this kind of result.

### 3.1. Hypotheses for Further Research

**Figure 6:**
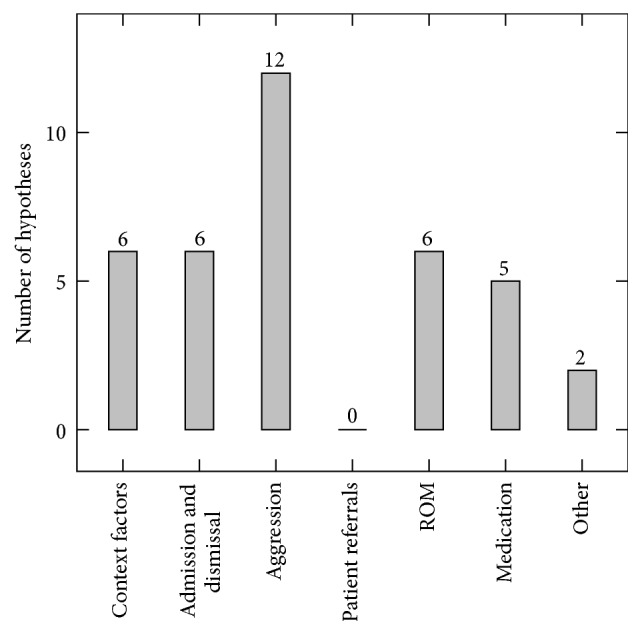
Number of hypotheses for further research found per theme, note that multiple themes can apply to one hypothesis.

**Figure 7:**
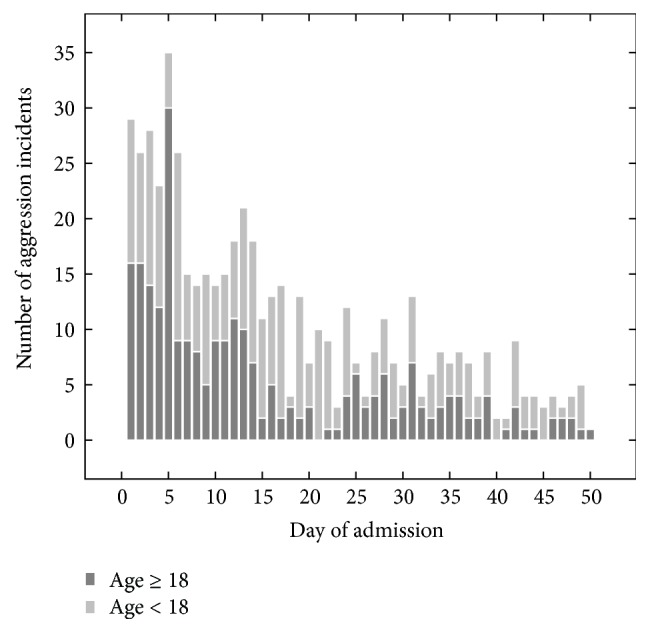
The total number of aggression incidents for the first 50 days of all admissions is visible (631 incidents). It can be seen that a peak occurs at day five, especially in adult patients (dark grey).

From the result step in the exploration process, a list of 29 hypotheses and topics for further investigation were identified. As opposed to the list of topics identified in Table 1, all of these assertions have some basis in the data. Of the 29 hypotheses, 5 are marked as directly related to one of the topics in Table 1; the other 24 have explicitly been found while exploring the data. This makes the proposal of hypothesis finding using interactive data analysis a success, since a large part of the found hypotheses are distinct from the questions that were known before looking at the data.  Figure 6 shows a breakdown of the number of found hypotheses per theme. Note that the Patient Referral theme was abandoned in the data understanding phase since obtaining data from other institutions was not viable within the duration of the project. It can be seen that most hypotheses pertained to the aggression theme, followed by context factors, admission and dismissal, Routine Outcome Monitoring, and medication themes with a roughly equal number of hypotheses each.

A top five of hypothesis for further investigation selected by health care professionals is displayed in Table 5. It is important to note that these hypotheses have a basis in data but for reasons mentioned above are not to be interpreted as direct results; for this additional research is required.

### 3.2. Process Evaluation

Carrying out the case study along our CRISP-IDM method generally went well, although some practical difficulties were experienced as well. Some of the most important findings for each of the phases are listed below:

1. *Domain Understanding*. Conducting expert interviews and identifying the seven research themes generally succeeded in getting acquainted with the research domain. The importance of this step cannot be underestimated, both because it forms the basis for the data selection and thereby directly influences the successfulness of the project and because it is the first step in interacting with local workforce.
2. *Data Understanding*. Some difficulties were experienced in obtaining the relevant data. The staff that supplies the data (e.g., data managers) may not be familiar with the idea of exploratory data analysis, so a clear statement of the project intentions is quite useful in order to obtain their support. Access to data was initially not very streamlined, because it was provided through several database systems and flat files that all have their own interfaces, methods, and file types. Yet eventually all necessary data was collected and centrally stored in a uniform format.
3. *General Data Preparation*. The steps to be taken in this phase are generally well-known; it is however time consuming to perform them on the data. The most commonly performed tasks are well described in literature, and applying them poses no real fundamental challenges, yet they are inevitable in transforming the raw data to analyzable data. Another aspect is that data quality deficits may come to light that are related to registration issues, and resolving those deficits requires inquiring with the people that are responsible for data registration, another time consuming task.
4. *Modeling and Evaluation*. Focusing on one theme during each of the sessions offered a way to balance between utilizing the diversity of the data and not overwhelming participants with it. A decent introduction to the visualization tool and its different visualizations proved to be effective, demonstrated by the fact that nearly all participants were able to contribute in the exploration sessions. The specific data preparation and visualization setup requires effort of technical staff but ultimately did not lead to significant challenges. The attitude of participants towards data analysis in this interactive manner was generally positive and open, which is further supported by the fact that during the later stages of the project the local professionals started taking initiative in asking questions that might be answered using the data outside the weekly sessions. This is in line with the ultimate goal of adopting a more data driven standard in the mental health institution.
5. *Deployment*. During the deployment phase, it turned out to be very helpful that health care professionals were strongly involved in the modeling and evaluation phase. The fact that they actively participated in finding the results led to a broad support in the daily work practice for implementing the outcomes.

## 4. Conclusion

The amount of data that has become available in health care enables conducting exploratory data analysis that focuses on finding new knowledge and hypotheses, instead of solving specific well-defined problems or testing existing hypotheses. We conducted a case study at the psychiatry department of the University Medical Center Utrecht to investigate the possibilities and limitations of this new exploratory type of data analysis. We furthermore actively involved local workforce in all steps of the process, both to guide and to strengthen the analysis and to pave the way for an implementation. For the case study, we have proposed a specification of the CRISP-DM that we named CRISP-IDM: Cross Industry Standard Process for Interactive Data Mining. In CRISP-IDM, most importantly the modeling and evaluation phases have been contracted into one phase that requires participation of health care professionals. Furthermore, the data preparation phase has been split in a general and specific preparation phase, and an optional inferential analysis step has been added after the modeling and evaluation phase.

During the domain understanding phase, expert interviews were conducted resulting in the identification and prioritization of seven research themes in the psychiatry department. In the subsequent data understanding and data preparation phases, suitable data for researching the themes was collected and general data preparation tasks such as transforming, cleaning, integrating, and enriching the data were performed. In the modeling and evaluation phase, data visualization was used as a tool to explore the data in collaboration with the health care professionals who guided the analysis in weekly sessions. Technical staff was responsible for selecting initial datasets, performing specific data preparation tasks that were discovered during the iterative exploration, and setting up the visualizations.

A total of 19 exploratory sessions were held with 18 different health care professionals, resulting in two direct results that were implemented in cooperation with both management and workforce professionals. Firstly, domain experts judged that the Kennedy Axis V scoring method was too constant to be of clinical use, and secondly strongly varying extensiveness of nursing reports initiated a new nurse report writing protocol. Furthermore, 29 hypotheses for further research were found, pertaining to six of the seven research themes that were identified in the domain understanding phase. Of these 29 hypotheses, 24 had not been imagined during the initial expert interviews. We have demonstrated the viability of using our CRISP-IDM method to organize exploratory and collaborative expert sessions and for effectively finding new knowledge and hypotheses.

## Figures and Tables

**Figure 8:**
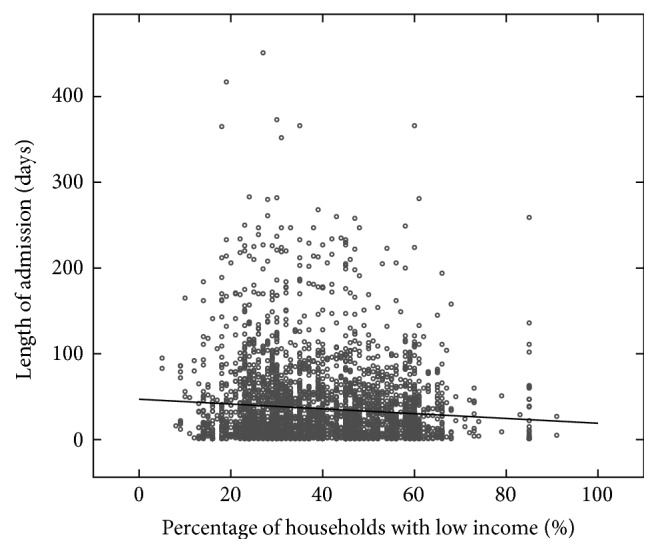
On the horizontal axis, the percentage of households with a low income in the neighborhood of a patient. On the vertical axis, the length of admission (2238 admissions). A trend is visible towards shorter admissions for patients from a neighborhood with a high percentage of low income households, that is, low economic status.

**Table 1:** The five identified topics with the highest priority, along with the theme they are categorized to. Note that these are not questions that must be answered during the project but serve as a way to become acquainted with the psychiatry department as a case study domain.

Topic	Theme	Priority
What are relations between the different Routine Outcome Monitoring (ROM) scores (or between specific sections of the ROM scores), and can they predict length of treatment?	Routine Outcome Monitoring	1
Do medication prescription and change in medication influence the length of admission and the likeliness of readmission?	Medication	2
Can aggression incidents in inpatients be predicted?	Aggression	3
In what way are patients referred between, for example, general practitioners, secondary care institutions, and the UMCU?	Patient referrals	4
What are descriptive statistics of aggression incidents? That is, in what location, at what time, and with which members of staff do they occur?	Aggression	5

**Table 2:** The seven themes that were derived from the topics that were mentioned in expert interviews (Table 1) and their priority.

Theme	Priority
Context factors	1
Admission & dismissal	2
Aggression	3
Patient referrals	4
Routine Outcome Monitoring	5
Medication	6
Other	7

**Table 3:** All acquired data entities and their sources, type, structuredness, and number of records (rounded to the nearest hundred or thousand).

Source	Data entity	Type	Structured/unstructured	Number of records
EPR	(1) Diagnosis	Categoric	Structured	5,800
	(2) Treatment plan	Categoric, textual	Both	6,500
	(3) Medication prescriptions	Categoric, numeric	Structured	22,000
	(4) Routine Outcome Monitoring	Numeric, textual	Both	13,000
	(5) Admission information	Categoric	Structured	5,400
	(6) Daily reports	Textual	Unstructured	150,000

Incident report system	(7) Aggression incident reports	Categoric, textual	Both	1,200

External	(8) Census data	Numeric	Structured	21,000
	(9) Geographic data	Numeric	Structured	5,000

**Table 4:** For each function, the total number of unique people involved and the total number of attendances in the interactive exploration sessions.

Main function	Number of people	Attendances
Psychiatrist	4	11
Psychologist	1	4
Nursing staff	4	9
Board	2	7
Policy maker	4	6
Other	3	4

*Total*	18	42

**Table 5:** Five of the hypotheses for further research selected by domain experts, in random order. Note that these hypotheses have some basis in data but need further research to turn into results. The second and fifth hypothesis are visualized in Figures 7 and 8.

Hypothesis	Theme
There exists a positive relation between season of admission and length of admission (longer admissions during winter)	Admission
A peak in aggression incidents occurs on the fifth day of admission (Figure 7)	Aggression
There exists a relation between aggression incidents and wearing of medication effects in patients diagnosed with ADHD	Aggression, medication
There is an absence of a relation between amount of green space in patient environment and likelihood of developing a disorder	Context factors
There is a negative relation between economic status of living environment and length of admission (Figure 8)	Admission, context factors
